# Is investing in the renewable energy stock market both financially and ESG efficient? A COVID-19 pandemic analysis

**DOI:** 10.1007/s11846-023-00664-7

**Published:** 2023-06-05

**Authors:** Amelia Bilbao-Terol, Mar Arenas-Parra, Raquel Quiroga-García, Celia Bilbao-Terol

**Affiliations:** 1 Faculty of Commerce Tourism and Social Sciences Jovellanos, University of Oviedo, Laboral Ciudad de la Cultura, 33203 Gijón, Spain; 2 Faculty of Business and Economics, University of Oviedo, Avda. Del Cristo S/N, 33006 Oviedo, Spain

**Keywords:** Data envelopment analysis, Financial efficiency, ESG efficiency, Energy stock market, COVID-19, G11, C61, 91B28, 91B06

## Abstract

The aim of this paper is to provide a tool for finding investments in the stocks of energy firms that achieve both good financial and reasonable environmental, social, and governance (ESG) performance. Our methodology entails two steps and is based on diversification-consistent DEA models. The first step involves constructing a financially efficient frontier of investment portfolios by applying the model originally proposed by Branda (Omega 52:65–76. 10.1016/j.ejor.2007.04.014, 2015). In the second step, a new DEA model is proposed in order to find the ESG-efficient portfolios among the ones already identified in the first step and to rank them with respect to their ESG performance. This model is parameterised by a weighting system that allows us to assign different importance to the various ESG outputs. Additionally, the proposal allows an evaluation of both ESG and financial efficiency related to the financial energy market over two periods (the pre-COVID-19 and COVID-19 periods), considering renewable energy and non-renewable energy firms both jointly and separately. The results support the better financial performance of the renewable energy stock market compared with that of the non-renewable energy market.

## Introduction

In recent years, data envelopment analysis (DEA), introduced by Charnes et al. (1978) has been applied and played an important role in many different areas of research, including energy (Mardani et al. 2017, 2018; Na et al. 2019; Dejian and Xiaorong 2020; Yu and He 2020) and finance (Lozano and Gutiérrez 2008; Perez-Gladish et al. 2013; Paradi and Zhu 2013; Lampe and Hilgers 2014; Premachandra et al. 2016; Basso and Funari 2016; Kaffash and Marra 2017; Bilbao-Terol et al. 2021) among others. Efficiency analysis provides information that helps decision-makers achieve better results.

Analysing a company’s environmental, social, and governance (ESG) performance as well as setting ESG goals and taking responsibility for achieving them has become an integral part of board agendas over recent years. There are numerous reasons why it is important to integrate ESG factors into investment decision-making. Several authors have suggested that companies are more likely to be successful and generate high returns if they create value for all their stakeholders—employees, customers, suppliers, and society in general, including the environment—and not just for the company (Harrison and Wicks 2013; Van der Linden and Freeman 2017; Signori et al. 2021). Analysis of ESG behaviour focuses on the service that companies provide to society and its effects on current and future results. Both conventional and socially responsible (SR) investors are concerned about the financial performance of their investments. For most SR investors, their investment in well-behaved ESG assets is not an act of charity. However, SR investors appear to have a greater acceptance of return spreads between conventional and screened investments, indicating that they derive utility from both the financial and non-financial characteristics of their investments. All these aspects imply that constructing a portfolio requires the appropriate treatment of the financial goals that both SR and conventional investors may have in mind.

The present paper has two aims. First, the relative financial and ESG efficiency of companies is assessed using two DEA models. Second, the financial (ESG) efficient frontier identified by the proposed models is used to obtain investment portfolios in the stocks of energy firms with ESG (financial) efficient performance. In this way, we provide a tool for finding investments that achieve both good financial and reasonable environmental, social and governance performance.

To test financial efficiency, we used Branda’s model (2015), which is consistent with second-order stochastic dominance (SSD). Hence, the expected rate of return was estimated by the output of the financial model under a finite number of equiprobable scenarios. A set of conditional risk values at several confidence levels were used as inputs of the model measuring financial performance. This approach allows investors to identify SSD-efficient portfolios. To determine the ESG efficiency of the investment portfolio, we propose a DEA model where it is assumed that all the inputs are the same for all firms.

The present study contributes to the existing literature in several ways. This is the first study to evaluate ESG efficiency using a DEA model containing weights associated with radial improvements of ESG outputs. The advantage of introducing weights in the modelling approach is twofold. Firstly, the investors can introduce their preferences in the DEA model. This means that the model provides efficient portfolios that are more adjusted to an investor’s preferences. Secondly, the parameterisation of the model via a weighting system allows the generation of more portfolios on the efficient frontier. The study also involves evaluating companies in the energy industry sector from both a financial standpoint (measured by their market return) and an ESG perspective (via public ESG ratings), which allows an assessment of their situation with respect to their competitors. In addition, a sequential and hierarchical methodology was proposed for investors with both financial and ESG goals. The sequence of applying the two models is determined by the investor’s profile. A conventional investor with ESG concerns could obtain their portfolio by first executing the financial DEA model and then applying the ESG model to the set of financially efficient portfolios. This study is also the first to analyse whether the COVID-19 pandemic has affected the financial and non-financial efficiency of a group of energy sector firms.

The rest of the paper is organised as follows. Section 2 presents a literature review, which is followed by a section that describes the two types of efficiency—financial and ESG efficiency—as well as their related DEA models. The following sections are devoted to a presentation of the empirical study. Our database consisted of 26 renewable and 52 non-renewable energy firms, which were analysed for the period 2018–2022. In addition, we considered two sub-periods (2018–2019 and 2020–2022) in order to analyse the influence that the COVID-19 pandemic may have had on the financial efficiency and ESG efficiency of the companies. The paper ends with the conclusions of the study.

## Literature review

Efficiency is a measure of the performance of a company that analyses the behaviour of its inputs and outputs over a certain period of time. Efficiency analysis provides information that will make it easier for company managers to establish programmes aimed at increasing a firm’s levels of competitiveness and productivity (Peng Wong and Yew Wong 2007).

Numerous studies have analysed the efficiency of companies in different economic sectors, both public and private (Emrouznejad and Yang 2018). In this context, several authors have provided overviews (both general and specific) of the DEA literature. Tavares (2002) presented a bibliography of DEA that consisted of 3,203 publications over the period 1978–2001. He also included an author and keyword index for the publications analysed. Liu et al. (2013) systematically surveyed DEA applications from 1978 through to August 2010.

The first published paper on the application of DEA to money market mutual funds was Murthi et al. (1997), who proposed a new DEA portfolio efficiency index to measure the performance of mutual fund portfolios. Since then, many papers have been published with different reformulations and emerging modifications of classical DEA models, mainly aimed at resolving problems such as the diversification phenomenon or the relationship between DEA efficiency and stochastic dominance. Lozano and Gutiérrez (2008) introduced several DEA-like linear programming models that are consistent with second-order stochastic dominance (SSD). Lamb and Tee (2012) proposed a stochastic DEA model based on a risk-return ratio for ranking funds. They discussed the relationship between diversification, coherent risk measures, and stochastic dominance. Branda (2015) extended the paper by Lozano and Gutiérrez (2008) by suggesting a new diversification-consistent DEA model equivalent to the SSD relationship using several risk measures as inputs and return measures as outputs, with both positive and negative values. Bilbao Terol et al. (2021) extended the DEA model of Branda (2015) to assess the overall efficiency of mutual funds, taking into account both financial and corporate sustainability characteristics.

A financial application of the DEA methodology is to gauge the efficiency of a company by using data from financial reports as inputs and outputs. For example, Edirisinghe and Zhang (2008) proposed a new approach based on DEA that combined financial data in order to develop a relative financial strength indicator to indicate stock price performance. They tested this indicator with US firms from the technology sector.

An important aspect that must be taken into account by companies is how they manage the impacts that their activity generates on their customers, employees, shareholders, local communities, the environment, and society in general. ESG performance measures a company against a set of ESG criteria in order to facilitate investment decisions. Today, interest in ESG issues has extended beyond investors to customers, employees, and other stakeholders. According to Whelan et al. (2021), the literature regarding the relationship between ESG and financial performance can be divided into two groups: those related to corporate financial performance, usually measured through different financial ratios, and those focused on investment performance, measured from an investor’s perspective through measurements of risk and return on assets or portfolios. Whelan et al. (2021) and Atz et al. (2021) analysed more than 1,000 papers in this field, and both studies found a positive relationship between ESG and financial performance at the corporate level. However, in relation to investment performance, their overall studies did not reveal a significant advantage for ESG investment, with returns from conventional investment strategies proving indistinguishable from ESG investment ones.

During the global economic recession following the subprime mortgage crisis, which particularly affected the financial markets, ESG investments performed better or as well as traditional investments. Numerous researchers have studied this effect to test whether this type of SR investment provides any kind of downside protection in times of crisis. Nofsinger and Varma (2014) stated that SR mutual funds improve the performance of conventional mutual funds during periods of market uncertainty. Fernández et al. (2019) found that green mutual funds in Germany outperformed conventional funds during the years of the 2007–2009 financial crisis. Wu et al. (2017) reported the same result in an analysis of the FTSE4Good index (formed by a set of ESG stock market indices). Similar results were found by Das et al. (2018) based on a Sharpe ratio study of the period 2005–2016, and they concluded that mutual funds with better ESG ratings outperformed those with lower ratings. As an explanation, Chatterjee et al. (2018) demonstrated that during years of greater market declines, funds with better ESG ratings presented better Sharpe ratios. Leite and Cortez (2018) pointed out that European socially responsible investing (SRI) funds were less exposed to bonds of the countries that were affected by the Euro sovereign debt crisis.

Since the COVID-19 pandemic, practitioners and researchers have speculated whether ESG investments could again prove a safe investment—or at least better than conventional ones—by providing downside protection similar to that detected during the financial crisis. For the European funds, Mirzaa et al. (2020) found that social entrepreneurship funds displayed resilience and performed better than non-social funds during the first half of 2020. Singh (2020) analysed the spillover effects of three different investment strategies during the pandemic crisis and demonstrated how capital rapidly took refuge in the ESG corporate index. These results support the importance of corporate fundamentals during a crisis: ESG companies are seen as being focused on long-term sustainability to attract investor attention during an economic downturn. Broadstock et al. (2021) also argued that investors may interpret ESG performance as a form of risk mitigation in periods of crisis and demonstrated the resilience of stocks with high ESG ratings in times of financial crisis in the Chinese market.

However, there is no consensus in the literature about the influence of ESG ratings on the performance of different financial assets. Studies such as Folger-Laronde (2020) (for ESG stocks) or Pavlova and de Boyre (2022) (for ESG exchange-traded funds) did not find evidence for high ESG ratings ensuring better performance during market downturns. Demers et al. (2021) found that the better performance of ESG stocks during the COVID-19 crisis was not due to their ESG rating, but rather the greater importance of each company’s investment in intangible assets.

Alongside the research analysing the performance of ESG assets, other studies have centred their attention on the financial resilience of companies. If we focus on the energy sector, one of the first studies was by Czech and Wielechowski (2021), who determined that the alternative energy sector appears to be more resilient than the conventional energy sector. They also concluded that this may be because the pandemic has increased interest in climate change and renewable energy. This idea was supported by the work of Wielechowski and Czech (2022) who analysed the period 2020–2021 to compare the profitability of the energy sector with other sectors, finding that, in general, energy sector companies provided the highest profitability. Lee (2021) examined the impact of environmental responsibility on the financial performance of 75 firms from the MSCI World Energy index over the period 2013–2017. He showed that environmental responsibility practices positively affected a firm’s financial performance. Liu et al. (2022) studied the influence of COVID-19 on three renewable energy stock indices from around the world. They found that economic uncertainty affected returns and, to a larger extent, the volatilities of renewable energy stocks.

The interest of individual and institutional investors in these types of investments has led to an increasing volume of academic literature on the development of methodologies based on mathematical programming for constructing portfolios tailored to the tastes and concerns of SRI investors. A pioneering work in this field was conducted by Hallerbach et al. (2004), which was based on the “New Approach to Consumer Theory” by Kelvin Lancaster (1966). According to this theory, utility does not derive directly from the consumption of goods but instead from the properties/characteristics they possess. In addition, there are several other papers on portfolio selection that take into account the ethical, social, and environmental factors highlighted by SRI. Some academics have tried to extend or complement the classic models of portfolio selection that were initially proposed by Markowitz (1952) (e.g., Drut 2010; Dorfleitner and Utz 2012) while other studies have been based on multi-criteria decision-making (e.g., Hallerbach and Spronk 2002; Hallerbach et al. 2004; Bilbao-Terol et al. 2016; Spronk et al. 2016; Jiménez et al. 2021). Multi-criteria decision analysis (Zeleny 1974) provides a framework for managing an investment portfolio in which the investment opportunities are described in terms of a set of attributes, with part of this set intended to capture and express the effects on society (Hallerbach et al. 2004; Bilbao et al. 2015).

Pedersen et al. (2021) summarised risk and return by the Sharpe ratio (SR) and showed that the investor’s problem with three characteristics (risk, return, and ESG) can be reduced to a trade-off between ESG and the SR. They computed the highest attainable Sharpe ratio for each level of ESG to obtain an ESG-SR frontier that is independent of investor preferences. Moreover, they showed the costs and benefits of responsible investing. The benefit of ESG information can be quantified as the resulting increase in the maximum SR (relative to a frontier based on only non-ESG information). The cost of ESG preferences can be quantified as the drop in the SR when choosing a portfolio with better ESG characteristics than those of a portfolio with maximum Sharpe.

In the present paper, we propose a DEA approach for constructing portfolios with ESG and financial goals. Two DEA models are considered for this: one in which we only consider financial characteristics and another in which the outputs are the ESG scores. Both models are presented in the following section.

## Methodology: DEA models for testing the firm efficiency

We consider a set of firms $\Upsilon = \left\{ {F_{i} ,\;i = 1,...,N} \right\}$. Each firm $F_{i}$ is described by its random rate of return, $r_{i}$, and its scores on the $P$ environmental, social and governance pillars determined by $ESG_{p} (F_{i} )$, $p = 1, \ldots ,P$. The set of investment possibilities, $\Pi$, that can be built from $N$ firms is $\Pi = \{ I = (x_{1} ,...,x_{N} ) \in IR^{N} |\sum\limits_{i = 1}^{N} {x_{i} } = 1,x_{i} \ge 0,i = 1,...,N\}$. Then, the random rate of return of investment, $I$, is $r(I) = \sum\limits_{i = 1}^{N} {r_{i} x_{i} }$.

In our framework, the production possibility set, $PPS(\Pi )$, can be defined by the result vectors corresponding to feasible investment according to $PPS(\Pi ) = \left\{ {\left( {E(I),Risk(I),ESG(I)} \right)|I \in \Pi } \right\}$, where $E(I) = E[r(I)]$ is the expected rate of return of investment $I$, $Risk(I) = \left( {risk_{1} (r(I)), \ldots ,risk_{K} (r(I))} \right)$ is a vector of $K$ coherent risk measures of $r(I)$, and $ESG(I) = \left( {ESG_{1} (I) = \sum\limits_{i = 1}^{N} {ESG_{1} (F_{i} )x_{i} } , \ldots ,ESG_{P} (I) = \sum\limits_{i = 1}^{N} {ESG_{P} (F_{i} )x_{i} } } \right)$ is a vector of the scores on the $P$ environmental, social and governance pillars of investment $I$.

### Financial-efficiency: SSD-efficiency DEA model

We handle the financial efficiency of any investment, and therefore of each firm, using the *second-order stochastic dominance* (*SSD*) of its random rate of return (Kopa and Chovanec 2008):

Let $X$ and $Y$ be two random variables with respective cumulative probability distributions functions $F_{X} (x)$ and $F_{Y} (x)$, then $X$ second-order stochastically dominates $Y$, $X \ge_{SSD} Y$, if $E_{{F_{X} }} [u(x)] \ge E_{{F_{Y} }} [u(x)]$ for all concave utility functions $u$ such that these expected values exist.

Therefore, we use the following definition of *second-order stochastic dominance efficiency*: a random variable $X$ is *SSD-efficient* if and only if there is no random variable that strictly dominates $X$ by *SSD*, i.e., there is no $Y$ such that $Y >_{SSD} X$. Otherwise, the variable $X$ is *SSD-inefficient*.

Following the results proposed by Kopa and Chovanec (2008), we identify *SSD* by *CVaR*.^1^ Two discretely distributed random variables, $X$ and $Y$, can be compared with strict *SSD* relation using *CVaRs* for a finite number of levels. From *S* equiprobable scenarios and with $\alpha_{k} = k/S$, $k \in \{ 0,1, \ldots ,S - 1\}$, *X* strictly dominates *Y* by *second-order stochastic dominance* if and only if $CVaR_{{\alpha_{k} }} (X) \le CVaR_{{\alpha_{k} }} (Y)$, $k \in \{ 0,1, \ldots ,S - 1\}$ with at least one strict inequality.

Taking into account the above, a given investment *I* is *financially efficient* if its rate of return $r(I)$ is *SSD-efficient*. Therefore, a firm $F_{i}$ is *financially efficient* if its rate of return $r_{i}$ is *SSD-efficient*. Otherwise, firm $F_{i}$ is *financially inefficient*.

Since $CVaR_{0} (X) = - E(X)$ and assuming *S* equiprobable scenarios for the distributions of the rate of return of the given firm $F_{i}$, the financial efficiency can be represented by the Koopmans-Pareto efficiency of the vector $\left( {E(F_{i} ), - CVaR_{1/S} (F_{i} ), \ldots , - CVaR_{(S - 1)/S} (F_{i} )} \right)$.

To identify whether an investment $I_{0}$ in the set $\Pi$ is *SSD-efficient* or *SSD-inefficient* we consider the following linear DEA model:

F-DEA $$\left. {\begin{array}{*{20}l} {\mathop {\min }\limits_{{}} \;z^{SSD} (I_{0} ) = t - \frac{1}{S - 1}\sum\limits_{k = 1}^{S - 1} {\theta_{k} } } \hfill \\ {s.t.} \hfill \\ {t + \varphi = 1,} \hfill \\ {\frac{1}{S}\sum\limits_{s = 1}^{S} {\sum\limits_{i = 1}^{N} {r_{is} } } y_{i} \ge t\;E[I_{0} ] + \varphi e(I_{0} ),} \hfill \\ {\xi_{k} + \frac{1}{S - k}\sum\limits_{s = 1}^{S} {u_{sk} } \le t\:CVaR_{k/S} (I_{0} ) - \theta_{k} \:d_{k} (I_{0} ),\;k = 1,...,S - 1,} \hfill \\ {u_{sk} \ge - \sum\limits_{i = 1}^{N} {r_{is} } y_{i} - \xi_{k} ,\;s = 1,...,S;\;k = 1,...,S - 1,} \hfill \\ {\sum\limits_{i = 1}^{N} {y_{i} } = t,} \hfill \\ {t,\varphi ,y_{i} ,\theta_{k} ,u_{sk} \ge 0.} \hfill \\ \end{array} } \right\}$$

where $e(I_{0} ) = \mathop {\max }\limits_{F \in \Upsilon } E[F] - E[I_{0} ]$ and $d_{k} (I_{0} ) = CVaR_{k/S} (I_{0} ) - \mathop {\min }\limits_{I \in \Pi } CVaR_{k/S} (I)$ are the non-negative directions, and $t,\varphi ,y_{i} ,\theta_{k} ,u_{sk} {\text{ and }}\xi_{k}$ are the decision variables (Branda 2015; Bilbao-Terol et al. 2021). We assume *S* equiprobable scenarios for the distribution of rates of return of the given firms with $r_{is}$ being the rate of return of $F_{i}$ for the scenario *S*. In this model the only output is the expected rate return and the inputs are the *S* $- 1$ *CVaRs*. The optimal objective value of the F-DEA model is the DEA score of $I_{0}$. If the DEA score is equal to 1, the investment $I_{0}$ is DEA-efficient, otherwise $I_{0}$ is DEA-inefficient.

### ESG-efficiency: ESG-DEA model

We define the e*nvironmental, social and governance efficiency* of an investment $I_{0}$ as:

$I_{0}$ is *ESG-efficient* if and only if there does not exist $I \in \Pi$ for which $ESG(I) \ge ESG(I_{0} )$ and $ESG(I) \ne ESG(I_{0} )$, i.e., $ESG_{p} (I) \ge ESG_{p} (I_{0} )$ for all ESG pillars with at least one strict inequality.

Therefore, the *ESG efficiency* of a firm $F_{i}$ can be represented by the Koopmans-Pareto efficiency of the vector $\left( {ESG_{1} (F_{i} ), \ldots ,ESG_{P} (F_{i} )} \right)$.

Next, we propose the following DEA model for determining the *ESG-efficiency* of the investment portfolio $I_{0}$:

ESG-DEA $$\left. {\begin{array}{*{20}l} {\max \;D^{ESG} (I_{0} ) = \sum\limits_{p = 1}^{P} {w_{p} \beta_{p} } } \hfill \\ {s.t.} \hfill \\ {\sum\limits_{i = 1}^{N} {ESG_{p} (F_{i} )\;x_{i} } \ge ESG_{p} (I_{0} ) + \beta_{p} \,f_{p} ,\;p = 1,...,P,} \hfill \\ {\sum\limits_{i = 1}^{N} {x_{i} } = 1,} \hfill \\ {\beta_{p} ,x_{i} \ge 0.} \hfill \\ \end{array} } \right\}$$

with the non-negative direction for each ESG pillar *p*: $f_{p} = \mathop {\max }\limits_{F \in \Upsilon } ESG_{p} (F)$ and decision variables $\beta_{p}$ and $x_{i}$; $w_{p} > 0$ being the weight associated with the ESG pillar *p*. We set $\sum {w_{p} } = 1.$

The weights in the objective function allow the modelling of investor preferences regarding the distances to the ESG outputs of the ESG-DEA model’s investment solution. In classical DEA framework the weights would be equal. A large weight assigned to pillar *p* rewards the movement factor up to $ESG_{p} (I^{*} )$ with $I^{*}$ being the investment solution of the ESG-DEA model. Therefore, the investment solution will tend to reach high values on pillar *p*. In addition, using different weight systems would allow new portfolios to emerge on the efficient frontier.

#### Proposition 1

An investment portfolio $I_{0} \in \Pi$ is *ESG-efficient* if and only if it is DEA-efficient according to the ESG-DEA model, i.e., $D^{ESG} (I_{0} ) = 0$.

#### Proof

First, it is possible to prove that each $\beta_{p}$ is less or equal than 1, assuming that before solving the DEA model, $\beta_{p}$ is set to 0 if $f_{p} = 0$.

Suppose $I_{0}$ is DEA-efficient according to the ESG-DEA model and *ESG-inefficient*. This means that there is an investment $I^{*} = \left( {x_{1}^{*} , \ldots ,x_{N}^{*} } \right) \in \Pi$ with $ESG_{p} (I^{*} ) \ge ESG_{p} (I_{0} )$ for all ESG pillars $p \in P$ with at least one strict inequality (say $q \in P$). Therefore, $D_{q} = ESG_{q} (I^{*} ) - ESG_{q} (I_{0} ) > 0$. In consequence, $f_{q}$ is not equal to zero and, therefore, it is possible to set $\beta_{q} = \frac{{D_{q} }}{{f_{q} }} > 0$. We also set $\beta_{p} = 0$ if $p \ne q$. Thus, there exists a feasible solution $\left( {(x_{i}^{*} ,\,\;i = 1,...,N),\;(\beta_{p} ,\,p = 1,...,P)} \right)$ for the ESG-DEA model with the objective value greater than 0. Hence, $I_{0}$ is DEA-inefficient. This is a contradiction.

Conversely, suppose $I_{0}$ is *ESG-efficient* and DEA-inefficient according to the ESG-DEA model; then, there exists $(x_{1}^{*} , \ldots ,x_{n}^{*} )$ a solution of the ESG-DEA model with $D^{ESG} (I_{0} ) > 0$. This implies that there is at least one $\beta_{p} > 0$ and, therefore, the investment $I^{*} = (x_{1}^{*} , \ldots ,x_{n}^{*} )$ verifies $ESG_{p} (I^{*} ) > ESG_{p} (I_{0} )$, which contradicts the *ESG-efficient* nature of $I_{0} .$

It is possible to prove that the optimal values of the ESG-DEA model are decreasing with respect to an ordering of the ESG characteristics of the investment portfolios, i.e., if an investment has higher ESG scores than another one, then it achieves a lower or equal DEA score in the ESG-DEA model.

#### Proposition 2

Consider $I_{1} ,\,I_{2} \in \Pi$. If $ESG(I_{1} ) \le ESG(I_{2} )$, then $D^{ESG} (I_{1} ) \ge D^{ESG} (I_{2} )$.

*Proof*. The hypothesis $ESG(I_{1} ) \le ESG(I_{2} )$ implies $ESG_{p} (I_{1} ) \le ESG_{p} (I_{2} )$ for all $p \in P$ ESG pillars. Let $\beta_{p}^{*} ,\,I^{*}$ be optimal solution of the ESG-DEA model with reference $I_{2}$. Then we obtain:

$$ESG_{p} (I^{*} ) \ge ESG_{p} (I_{2} ) + \beta_{p}^{*} \,f_{p} \ge ESG_{p} (I_{1} ) + \beta_{p}^{*} \,f_{p}$$

Therefore, $\beta_{p}^{*} ,\,I^{*}$ is feasible for the ESG-DEA model with reference $I_{1} .$ Hence, since the ESG-DEA model is a maximisation problem, the optimal value for $I_{1}$ is greater than or equal to the one for $I_{2}$: $\,\,D^{ESG} (I_{1} ) \ge D^{ESG} (I_{2} )$.

Analogously to the property of the F-DEA model, it is possible to prove that the portfolio solution of the ESG-DEA model is efficient with respect to this model and, in consequence, applying Proposition 1, this portfolio is ESG-efficient.$\square$

#### Proposition 3

Let $\beta_{p}^{*} ,\,I^{*}$ be the optimal solution of the ESG-DEA model for a reference $I_{0} \in \Pi$. Then, the portfolio $I^{*}$ is efficient with respect to this model.

#### Proof

We suppose $I^{*}$ is not efficient with respect to the ESG-DEA model, i.e., $D^{ESG} (I^{*} ) > 0.$ Then, there is an investment portfolio, *I*, verifying $ESG_{p} (I) \ge ESG_{p} (I^{*} ) + \beta_{p} f_{p}$ for all pillars, *p*, with at least one $\beta_{q} > 0$. Since $I^{*}$ is the optimal solution of the ESG-DEA model for reference $I_{0} \in \Pi$, then it is verified $ESG_{q} (I^{*} ) + \beta_{q} f_{p} \ge ESG_{q} (I_{0} ) + \beta_{q}^{*} \,f_{q} + \beta_{q} \,f_{q}$. Therefore, *I* is a feasible solution for the ESG-DEA model with a reference $I_{0}$ which achieves a higher value $\left( {\beta_{q} + \beta_{q}^{*} } \right)$ of the objective function than $D^{ESG} (I^{*} )$. This is a contradiction.$\square$

In this section, we introduced the DEA models employed for identifying the *financial efficiency* and *ESG efficiency* of an investment portfolio $I_{0} \in \Pi$. Table 1 describes the inputs and outputs included in each model. In order to unify the scale, we set $1 - D^{ESG} (I_{0} )$ as the DEA score of the ESG-DEA model.

**Table 1:** Inputs/outputs for DEA Models

DEA Models	Inputs	Outputs
F-DEA model	Coherent Risk Measures:$CVaR_{k/S} (I_{0} ),k = 1,...,S - 1$	Expected Rate of Return:$E[I_{0} ]$
ESG-DEA model		ESG scores:$ESG_{p} (I_{0} ),\;p = 1,...,P$

Efficient portfolios associated with each firm were obtained from the F-DEA and ESG-DEA models. For those firms that are efficient, their associated portfolio consists of the firm itself. For non-efficient firms, an efficient portfolio was obtained consisting of companies from the investment universe.

## Case study data: the energy industry

The financial and ESG data for this paper come from the Refinitiv database. This database is one of the world’s largest providers of financial market data and infrastructure. The fundamental financial performance of a firm is closely related to its stock price performance. We considered the weekly stock prices for each company and calculated the weekly logarithmic returns. The weekly stock prices, as well as the ESG scores, were checked for completeness and only those firms with complete data were chosen. The sector chosen as the focus of the study was the energy sector: renewable and non-renewable energy firms. Table 2 shows the filters that were used to select the renewable energy firms that form part of our database.

**Table 2:** Refinitiv filters

Currency	Euro (EUR)
Universe	Public Companies
Country of Exchange	Asia, Europe, Africa, Americas, Oceania
TRBC Industry name	Renewable energy Equipment & services (269) Renewable Fuels (111)
ESG Score	> 0.01 (2018, 2019, 2020). Total = 26 firms

After applying these filters, we were left with 26 firms included in the Refinitiv business sector of *Renewable Energy*. By region, there were 13 firms in America, seven in Europe and six in Asia.^2^ In order to evaluate the impact of being a renewable energy firm, we needed to analyse energy firms both with and without the ‘renewable’ label. To select the set of non-renewable energy companies, a matching methodology was applied (see, e.g., Ho et al. 2007; Stuart 2010, and references therein for further details). We conducted a 2:1 nearest neighbour matching with a logistic regression-based propensity score, which resulted in 52 non-renewable energy companies matched with the 26 renewable energy ones. The variables ‘country of exchange’ and ‘market capital’ were used as covariates in the matching process. Therefore, our final database had 78 firms: Firm 1 to Firm 26 correspond to renewable energy companies and Firm 27 to Firm 78 correspond to non-renewable ones.^3^

Refinitv collects ESG data from publicly available sources and from companies’ public disclosure (annual reports, company websites, NGO websites, stock exchange filings, CSR reports, and news sources). This database has more than 150 content research analysts trained to collect more than 400 ESG measures across the globe. All the collected information is divided into three pillars, ‘environmental’, ‘social’, and ‘governance’, that, in turn, include different categories and components (see Table 19 in the Appendix). The ESG scores vary on a scale from 0 to 100.^4^

The analysed period was divided into two sub-periods: the pre-COVID-19 period (1/1/2018–12/31/2019) and the COVID-19 period (1/1/2020–2/28/2022). A summary of the expected return (ER), *CVaR* at 95% confidence level ($CVaR_{95}$), and ESG scores for both renewable and non-renewable energy firms are presented in Tables 3 and 4 for the pre-COVID-19 and COVID-19 periods, respectively. It can be seen that the mean financial values and the mean environmental and social scores are better for the renewable energy companies than for the non-renewable ones in both periods. The mean scores in ‘governance’ are slightly better for non-renewable energy companies in both periods. However, the maximum values are mostly reached by non-renewable energy companies.

**Table 3:** Summary of the company data for the pre-COVID-19 period

			2018			2019		
	ER	$CVaR_{95}$	ENV Score	SOC Score	GOV Score	ENV Score	SOC Score	GOV Score
Renewable energy firms								
Minimum	− 0.02105	0.07491	0.00000	5.69444	9.59302	0.00000	3.88258	10.25773
Mean	0.00064	0.15262	42.21431	46.69297	44.08976	47.07430	49.73625	43.48699
Max	0.02516	0.26238	81.32082	85.89744	88.26190	81.76176	89.33363	81.09788
Non-renewable energy firms								
Minimum	− 0.02072	0.06139	0.00000	2.76541	9.78983	0.00000	2.66009	6.90196
Mean	− 0.00346	0.15549	23.23611	31.33264	44.76922	26.76806	33.33795	47.32594
Max	0.01184	0.27171	90.26681	87.92708	85.43132	88.36990	90.88474	89.82956

**Table 4:** Summary of the company data for the COVID-19 period

			2020		
	ER	$CVaR_{95}$	ENV Score	SOC Score	GOV Score
Renewable energy firms					
Minimum	− 0.01878	0.10758	0.00000	12.90850	22.30037
Mean	0.00569	0.19445	49.33053	57.71550	49.08899
Max	0.01922	0.29015	80.53461	88.72290	82.75852
Non-renewable energy firms					
Minimum	− 0.01217	0.08485	0.00000	4.37040	9.18699
Mean	− 0.00083	0.24306	30.78863	36.86219	51.39882
Max	0.01897	0.50326	90.10564	90.47876	94.09100

If we compare the two periods, we observe that the maximum value of the expected return corresponds to the pre-COVID-19 period for renewable energy companies. However, if we look at the mean and the minimum, better values are obtained for the COVID-19 period. Moreover, non-renewable energy companies are more profitable in the COVID-19 period (see column 1, Tables 3 and 4). Regarding risk, we observe a higher risk in the COVID-19 period for both renewable and non-renewable energy companies (see column 2, Tables 3 and 4).

## Empirical results and discussion

**Fig. 1:**
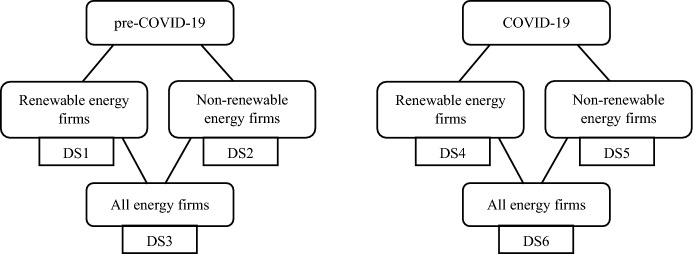
Data sets for the different periods

In this section, we review the main results obtained in the empirical analysis, after applying the two DEA models (F-DEA and ESG-DEA) to the different periods and data sets (see Fig. 1).

### Financial-DEA model for the energy firms

The Financial-DEA model (F-DEA) was applied to six different data sets: all the energy companies jointly and renewable and non-renewable energy firms separately, for the pre-COVID-19 and COVID-19 periods. Table 5 shows a summary of the F-DEA scores. In all cases, only one firm was rated as efficient, corresponding to the one with the highest expected profitability. F-DEA is very demanding as it uses a large number of *CVaRs* (101 and 111 in the pre-COVID-19 and COVID-19 periods, respectively) and it is difficult for just one company to achieve efficient diversification. When considering the whole set of firms, the only efficient firm belonged to the renewable energy subsector. There was an increase in average financial efficiency of 28%, 25.3%, and 50.2% during the COVID-19 period compared to the pre-COVID-19 period in the sets of all firms, only renewable energy firms, and only non-renewable energy firms, respectively (see Table 5).

**Table 5:** Summary of F-DEA-scores for the energy firms

	Renewable firms	Non-renewable firms	All firms
Pre-COVID-19 Period			
Minimum	0.02482	0.01746	0.02573
Mean	0.12146	0.08244	0.08791
Max	1	1	1
Efficient firms	Firm 22	Firm 44	Firm 22
COVID-19 Period			
Minimum	0.02976	0.02955	0.03485
Mean	0.15223	0.12384	0.11250
Max	1	1	1
Efficient firms	Firm 17	Firm 27	Firm 17

For the pre-COVID-19 period, the only renewable energy company rated as efficient was Firm 22, which was also the only efficient company when considering all firms together. Firm 22, Enphase Energy, Inc. (ENPH.OQ), is an energy technology company. The company is a supplier of microinverter-based solar and battery systems. After a sustained growth in its stock closing price from 01/12/2018 to 07/26/2019, its closing price increased substantially on 08/02/2019. Considering the pre-COVID-19 period, the first closing price was €1.84 and the last was €23.94. Its $CVaR_{95}$ was equal to 0.206; therefore, according to Table 3, it could be considered a risk stock. Regarding the non-renewable energy subsector, Firm 44 was the only efficient company. Firm 44 is PrimeEnergy Resources Corporation (PNRG.OQ), an independent US oil and gas company that is engaged in acquiring, developing, and producing oil and natural gas. Its stock price had similar behaviour to that of Firm 22, starting from €40.38 and increasing substantially on 08/02/2019 to reach €135.12 by the end of 2019. A $CVaR_{95}$ value of 0.117 places this company as lower risk than Firm 22.

For the COVID-19 period, the only efficient renewable energy firm was Firm 17, GCL Technology Holdings Ltd (3800.HK), which is a Hong Kong investment holding company mainly engaged in the manufacture and sale of solar materials. It achieved the highest expected return. Its $CVaR_{95}$ was equal to 0.187 and is therefore potentially classifiable as medium risk according to Table 4. Of the non-renewable energy companies, the only efficient firm was the Firm 27, Antero Resources Corporation (AR.N), which is an independent U.S. oil and natural gas company. The company is engaged in the development, production, exploration, and acquisition of natural gas, natural gas liquids, and oil resources located in the Appalachian Basin. Its closing prices increased almost tenfold during the COVID-19 period. However, its risk is quite high with a $CVaR_{95}$ of 0.228, which certainly prevents it from being rated as efficient when considering all the companies simultaneously.

### ESG-DEA model for the energy firms

#### ESG-DEA model with equal weights

When we applied the ESG-DEA model with equal weights for the three ESG pillars, we observed an increase in average ESG efficiency of 17.39%, 9.52%, and 18.36% during the COVID-19 period compared to the pre-COVID-19 period in the sets of all firms, only renewable energy firms, and only non-renewable energy firms, respectively (see Table 6). In contrast to what happened when studying financial efficiency, in which only one company was efficient in each of the analysed cases, between four and seven companies were rated as efficient when looking at ESG efficiency, depending on the case. In the pre-COVID-19 period, among the ESG-efficient companies obtained for the case of renewable energy firms (DS1), only Firm 3 remained efficient when considering all firms together. However, all the ESG-efficient firms obtained for non-renewable energy firms, are still rated as ESG-efficient when considering all firms jointly. In the COVID-19 period, the ESG-efficient firms obtained in the ESG-DEA models for non-renewable energy firms (DS5) and all firms (DS6) coincided. In this period, no renewable energy firm was rated as ESG efficient when considering all firms together since most of the maximum values of the ESG pillars were reached by non-renewable energy firms. Note that financially efficient firms do not appear as efficient when analysing ESG efficiency in both periods (see Tables 5 and 6). This is because financially efficient firms have poor ESG behaviour, which can be seen when compared to their associated efficient ESG portfolios (see Table 20 in the Appendix).

**Table 6:** Summary of ESG-DEA scores for the energy firms

	Renewable firms	Non-renewable firms	All firms
Pre-COVID-19 Period			
Minimum	0.25512	0.10257	0.10604
Mean	0.64670	0.46401	0.50228
Max	1	1	1
Efficient firms	Firm 3, Firm 5, Firm 7, Firm 12	Firm 30. Firm 45, Firm 54, Firm 67, Firm 75, Firm 76	Firm 3, Firm 30. Firm 45, Firm 54, Firm 67, Firm 75, Firm 76
COVID-19 Period			
Minimum	0.31069	0.19989	0.19989
Mean	0.70827	0.54916	0.58964
Max	1	1	1
Efficient firms	Firm 1, Firm 3, Firm 12, Firm 21, Firm 23	Firm 30. Firm 45, Firm 53, Firm 54, Firm 67	Firm 30. Firm 45, Firm 53, Firm 54, Firm 67

By comparing the same set of firms across the two periods, we can observe that only two renewable energy firms maintained their ESG efficiency (Firm 3 and Firm 12) as well as three non-renewable energy firms (Firm 30, Firm 45, and Firm 54). Finally, when analysing all firms together, Firm 30, Firm 45, Firm 54, and Firm 67 maintained their ESG efficiency.

#### ESG-DEA model with different weights for renewable energy firms in the COVID-19 period

The ESG-DEA model was applied with different weights for the ESG pillars and for renewable energy firms in the COVID-19 period (see Table 7). The weights obtained by applying an extended best–worst method (Bilbao et al. 2022) were 0.077 for the ENV pillar, 0.165 for the SOC pillar, and 0.758 for the GOV pillar. In this case, the governance pillar was the most important and the environmental pillar the least important.

**Table 7:** Summary of ESG-DEA scores

Statistics	Values
Minimum	0.34159
Mean	0.70115
Max	1
Efficient firms	Firm 1, Firm 3, Firm 12, Firm 21, Firm 23

#### Analysis of the ESG efficient portfolios for renewable energy firms in the COVID-19 period

A comparison was carried out between the obtained results from the ESG-DEA with equal and different weights for renewable energy firms in the COVID-19 period. Naturally, both models rate the same firms as efficient, although the DEA scores of the remaining companies vary slightly. In order to show the usefulness of the proposed ESG-DEA model, we analysed the ESG-efficient portfolios obtained for the two analysed cases.

The ESG scores of the ESG-efficient portfolios are displayed in Tables 8 and 9, respectively. The results show how changing the importance of the different ESG pillars gives rise to changes in ESG-efficient portfolios. The equal weights provided six efficient portfolios. On the other hand, the different weights provided twelve efficient portfolios; therefore, six new portfolios appeared (portfolios associated with firms 5, 9, 14, 15, 18, and 24).

**Table 8:** ESG scores for ESG-efficient portfolios with equal weights

ESG-efficient portfolios	ENV score	SOC score	GOV score
P1	17.167	12.908	82.759
P2 to P10, P13 to P20, P22, P24 to P26	80.535	80.267	76.137
P11	80.520	80.277	76.121
P12	68.425	88.723	62.460
P21	77.755	88.597	39.483
P23	59.214	66.726	81.305

**Table 9:** ESG scores for ESG-efficient portfolios with different weights (0.077, 0.165, 0.758)

ESG-efficient portfolios	ENV score	SOC score	GOV score
P1	17.167	12.908	82.759
P2, P4, P6, P7, P8, P10, P13, P16, P17, P19, P20, P22, P23, P25, P26	59.214	66.726	81.305
P3	80.535	80.267	76.137
P5	79.780	79.788	76.320
P9	71.470	74.510	78.334
P11	80.520	80.277	76.121
P12	68.425	88.723	62.460
P14	64.807	70.278	79.949
P15	69.888	73.505	78.718
P18	68.307	72.501	79.101
P21	77.755	88.597	39.483
P24	76.298	77.576	77.164

For example, for Firm 2, the GOV-score proved to be higher than in the case DEA2-DS4. The same occurred for the portfolios associated with firms 4 to 10, firms 13 to 20, Firm 22, and firms 24 to 26.

### Analysis of the financial efficient portfolios

**Fig. 2:**
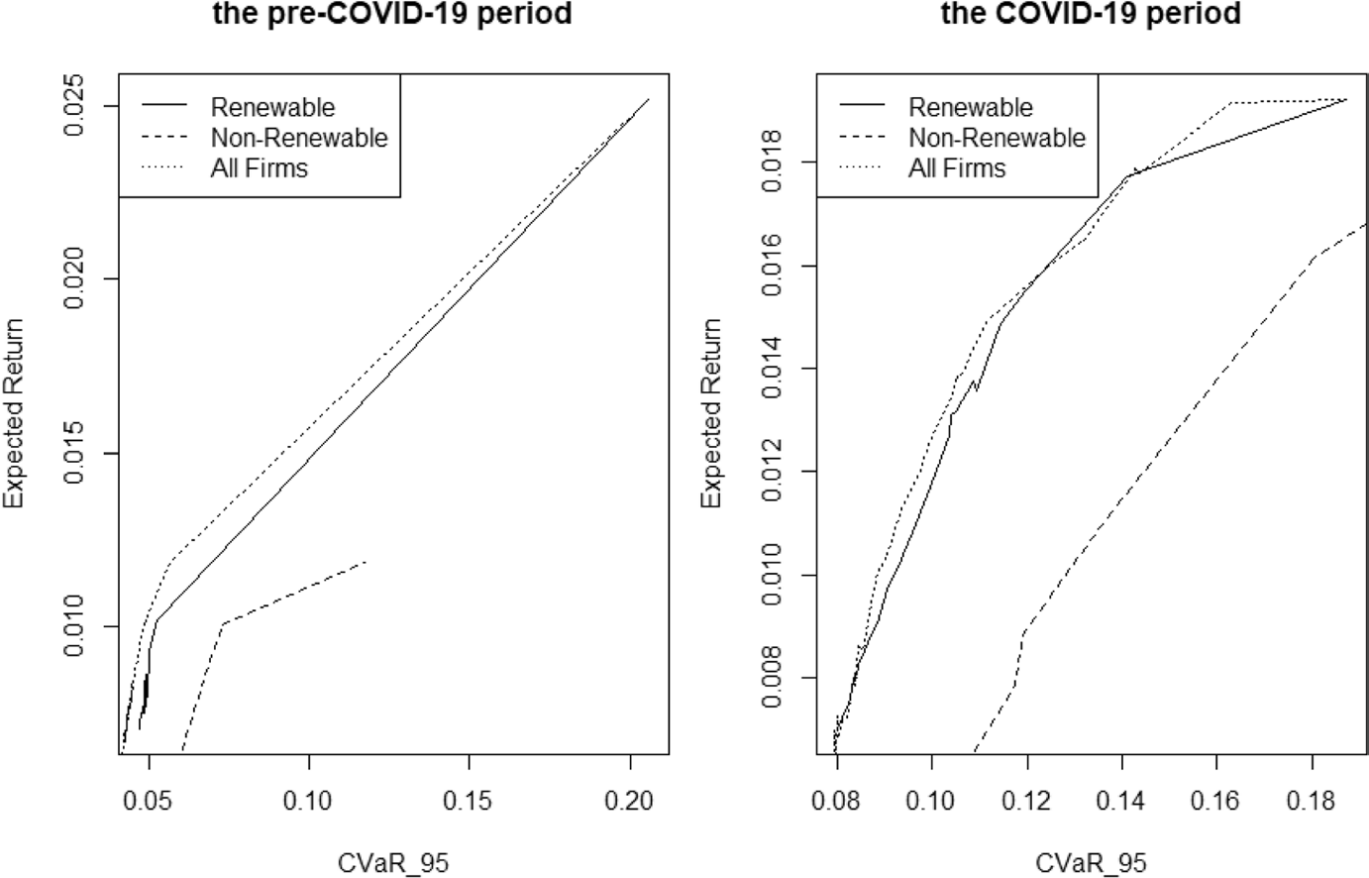
Efficient frontier for the pre-COVID-19 and COVID-19 periods

Based on the F-DEA model applied to the six data sets (DS1 to DS6), 26, 52, and 78 efficient portfolios were obtained in each case, respectively. Table 10 reports the descriptive statistics for these portfolios. The mean expected return of financially efficient portfolios obtained with exclusively renewable energy companies was higher than that obtained with only non-renewable energy companies. This result can also be seen in the efficient frontier for the pre-COVID-19 and COVID-19 periods shown in Fig. 2.

**Table 10:** Summary of financially efficient portfolios

	Renewable energy firms		Non-renewable energy firms		All energy firms	
	ER	$CVaR_{95}$	ER	$CVaR_{95}$	ER	$CVaR_{95}$
Pre-COVID-19 period						
Minimum	0.00707	0.04704	0.00257	0.04895	0.00633	0.04161
Mean	0.00875	0.05498	0.00381	0.05336	0.00813	0.04646
Max	0.02516	0.20592	0.01184	0.11720	0.02516	0.20592
COVID-19 period						
Minimum	0.00700	0.07996	− 0.00106	0.06779	0.00336	0.06378
Mean	0.01122	0.10099	0.00144	0.08329	0.00804	0.08671
Max	0.01922	0.18728	0.01897	0.22807	0.01922	0.18728

As can be seen in Fig. 2, the efficient frontier obtained when all firms were considered overlaps almost completely with the efficient frontier obtained from renewable energy firms only. To substantiate this result, we analysed the composition of the financially efficient portfolios obtained from the set of companies (DS3 and DS6). Table 11 shows the percentage of renewable energy companies in each efficient portfolio. For both the pre-COVID-19 and COVID-19 periods, the efficient portfolios were mostly made up of renewable energy companies: in the pre-COVID-19 period, 87% of the portfolios were made up of more than 50% renewable energy companies, and this percentage was 97% for the COVID-19 period. Moreover, in the pre-COVID-19 period, renewable energy companies represented 54% of the efficient portfolio on average, which increased to almost 63% in the COVID-19 period.

**Table 11:** Percentage of renewable energy firms in each efficient portfolio obtained from F-DEA for DS3 and DS6

Portfolio	pre-COVID-19 (%)	COVID-19 (%)	Portfolio	pre-COVID-19 (%)	COVID-19 (%)	Portfolio	pre-COVID-19 (%)	COVID-19 (%)
1	51.22	56.25	27	50.67	75.12	53	54.03	62.39
2	63.17	69.90	28	52.44	50.82	54	52.19	63.37
3	51.36	61.32	29	49.48	56.46	55	53.55	57.85
4	56.52	74.03	30	48.63	48.11	56	57.31	61.25
5	53.37	58.74	31	51.22	57.08	57	51.94	64.64
6	57.54	85.53	32	51.08	61.53	58	50.41	55.27
7	51.04	49.92	33	54.03	81.43	59	54.90	76.27
8	51.45	74.52	34	59.40	53.52	60	49.73	59.47
9	49.39	85.58	35	53.81	72.68	61	50.30	52.85
10	53.46	85.87	36	51.98	55.84	62	55.74	55.98
11	52.20	72.59	37	59.69	60.83	63	50.65	62.21
12	52.02	57.60	38	50.89	86.83	64	51.26	59.97
13	55.05	81.25	39	51.29	85.71	65	52.04	54.81
14	51.49	55.00	40	53.69	54.66	66	52.65	52.31
15	63.43	67.30	41	60.98	51.51	67	49.77	60.30
16	59.12	57.14	42	51.76	64.55	68	51.56	60.18
17	49.85	100.00	43	50.19	79.65	69	55.33	52.88
18	52.42	55.96	44	61.15	52.87	70	51.33	66.07
19	53.17	53.36	45	48.29	50.54	71	51.00	51.81
20	55.18	85.71	46	53.19	52.11	72	46.30	50.97
21	59.89	79.00	47	51.26	64.52	73	51,50	56.14
22	100.00	83.41	48	54.17	53.47	74	60.55	51.04
23	62.98	84.59	49	54.22	52.46	75	47,37	52.17
24	63.22	73.34	50	52.48	58.03	76	50.94	56.38
25	49.37	50.68	51	53.41	52.96	77	50.41	50.27
26	54,67	55,53	52	62,07	53,34	78	51.65	54.31

	Pre-COVID-19	COVID-19
Mean proportion renewable energy firms	54.08%	62.84%
Percentage times proportion renewable energy firms > 50%	87.18%	97.44%

The importance of the renewable energy companies 22 and 17 was reaffirmed when we analysed the distribution of the firms on the efficient financial frontier (Table 12) since they appeared in 78 efficient portfolios in the pre-COVID-19 and COVID-19 periods, respectively. We also want to highlight that out of all the companies that appeared in some proportion of the efficient portfolios in the first period, there were eight that appeared in 77 efficient portfolios (Firm 2, Firm 3, Firm 15, Firm 16, Firm 20, Firm 23, Firm 41, and Firm 44), six of which were renewable energy firms. For the COVID-19 period, we observed 20 companies that participated in some proportion of the efficient portfolios. Finally, the companies that appeared as part of an efficient portfolio in both periods, although with different participation percentages, were Firm 3, Firm 13, Firm 16, Firm 20, Firm 30, Firm 41, and Firm 61—more than 57% of which being renewable energy firms.

**Table 12:** Distribution of the firms on the financial efficient frontier

pre-COVID-19 period (DS3)				COVID-19 Period (DS6)			
Firm	Average of participation in portfolios (%)	Number of times	Frequency (%)	Firm	Average of participation in portfolios (%)	Number of times	Frequency (%)
2	5.92	77	98.72	3	1.50	55	70.51
3	16.53	77	98.72	6	8.07	74	94.87
13	0.23	28	35.90	8	4.94	76	97.44
15	6.12	77	98.72	9	10.70	74	94.87
16	5.67	77	98.72	13	17.82	76	97.44
20	1,50	77	98.72	16	7.11	72	92.31
21	0.50	24	30.77	17	9,13	78	100.00
22	7.67	78	100.00	18	7.41	62	79.49
23	9.57	77	98.72	19	1.22	9	11.54
25	1.82	62	79.49	20	0.43	15	19.23
30	5.84	69	88.46	27	4.93	69	88.46
41	11.52	77	98.72	30	17.35	58	74.36
44	18.72	77	98.72	41	1.75	8	10.26
45	2.58	48	61.54	43	0.39	1	1.28
61	4.33	71	91.03	49	1.17	45	57.69
73	0.51	8	10.26	55	1.55	55	70.51
75	5.75	72	92.31	60	1.19	59	75.64
				61	0.12	1	1.28
				77	16.39	65	83.33
				78	3.65	72	92.31

### Comparison of investors' profiles

Our proposal provides a tool to model two investor profiles. The first is a financially efficient investor who searches among financial efficient portfolios to identify those that are ESG efficient (F-ESG investor). The second is a socially responsible investor who searches among ESG efficient portfolios to identify those that are financial efficient (ESG-F investor).

#### F-ESG investor: ESG-DEA efficiency on the financial frontier

For the financially efficient portfolios obtained when considering all the companies simultaneously for the pre-COVID-19 and COVID-19 periods, each ESG-DEA efficiency was studied to observe their behaviour during the study period. The results are summarised in Table 13 and indicate that the ESG efficiency of the efficient frontier decreased during the COVID-19 period. This application of the ESG DEA model offered an ESG ranking of the financially efficient portfolios. The characteristics and composition of the ESG efficient portfolios obtained are shown in Tables 14 and 15.

**Table 13:** Summary of ESG-DEA scores of the financial efficient frontier

	Pre-COVID-19 period	COVID-19 period
Minimum	0.69936	0.71721
Mean	0.92188	0.90905
Max	1	1
Efficient portfolios	P22, P72	P30

**Table 14:** ESG-efficient portfolios on the financial efficient frontier over the pre-COVID-19 period

Portfolio	ENV 2018	SOC 2018	GOV 2018	ENV 2019	SOC 2019	GOV 2019	ER	$CVaR_{95}$
P22	34.953	58.141	32.149	33.204	57.312	18.341	0.025	0.206
	Composed of: Firm 22 (1)							
P72	39.556	46.570	45.678	43.110	48.942	46.520	0.006	0.042
	Composed of: Firm 2 (0.35), Firm 3 (0.148), Firm 15 (0.044), Firm 16 (0.062), Firm 20 (0.002), Firm 22 (0.048), Firm 23 (0.090), Firm 25 (0.034), Firm 30 (0.095), Firm 41(0.103), Firm 44 (0.150), Firm 45 (0.055), Firm 61 (0.061), Firm 73 (0.014), Firm 75 (0.060)

**Table 15:** ESG-efficient portfolio on the financial efficient frontier over the COVID-19 period

Portfolio	ENV 2020	SOC 2020	GOV 2020	ER	$CVaR_{95}$
P30	61.003	60.323	48.998	0.004	0.075
	Composed of: Firm 3 (0.018), Firm 6 (0.043), Firm 8 (0.044), Firm 9 (0.081), Firm 13 (0.093), Firm 16 (0.073), Firm 17 (0.032), Firm 18 (0.096), Firm 30 (0.277), Firm 49 (0.012), Firm 55 (0.029), Firm 60 (0.012), Firm 61 (0.001), Firm 77 (0.160), Firm 78 (0.028)				

In the pre-COVID-19 period, the F-ESG investor could choose between the portfolio P22 and the portfolio P72 (Table 14). Portfolio 22 consists of only Firm 22, the most profitable and high-risk firm. This portfolio has a slightly higher mean social score in both years (2018 and 2019). On the other hand, portfolio P72 (associated with the F-inefficient Firm 72) is a diversified low-risk portfolio composed of 15 firms. The largest share is for Firm 44 that jointly with the share of Firm 3 represents 30% of the portfolio P72.

During the COVID-19 period, the F-ESG investor could choose portfolio P30 (associated with the F-inefficient Firm 30). This portfolio is diversified, with 15 firms appearing, the largest share being that of Firm 30 which jointly with the share of Firm 77 represents 44% of the portfolio P72. With regard to its financial characteristics, it could be considered as medium profitable and low risk. Regarding its ESG characteristics, P30 achieves ENV and SOC scores above the mean and its GOV score is slightly below the mean (see Table 4).

It is noted that P72 and P30 are very different portfolios with respect to their compositions but both are investments incorporating low risk and low profitability.

#### ESG-F investor: F-DEA efficiency on the ESG efficient frontier

We have applied the F-DEA model to the portfolios of the ESG efficient frontier obtained by applying the ESG-DEA model to the whole energy stock market for the pre-COVID-19 and COVID-19 periods. The summary of results is displayed in Table 16. Tables 17 and 18 show the portfolios that are financial efficient on the ESG efficient frontier. Comparison between Tables 14 and 17 shows that the ESG scores obtained increase, even doubling their value, although accompanied by the trade-off of a significant drop in profitability levels.

**Table 16:** Summary of F-DEA scores on the ESG efficient frontier

	pre-COVID-19 Period	COVID-19 Period
Minimum	0.00723	0.00335
Mean	0.04911	0.18137
Max	1	1
Efficient portfolios	P3	P1, P3, P23, P30, P31, P37, P38, P42, P54, P62, P71, P76, P78

**Table 17:** Financial-efficient portfolio on the ESG efficient frontier over the pre-COVID-19 period

Portfolio	ENV 2018	SOC 2018	GOV 2018	ENV 2019	SOC 2019	GOV 2019	ER	$CVaR_{95}$
P3	73.812	84.551	88.262	73.975	82.933	63.520	0.005	0.075
	Composed of: Firm 3 (1)

**Table 18:** Financial-efficient portfolios on the ESG efficient frontier over the COVID-19 period

Portfolio	ENV 2020	SOC 2020	GOV 2020	ER	$CVaR_{95}$
P31	44.734	40.722	90.968	0.001	0.256
	Composed of: Firm 30 (0.171), Firm 54 (0.829)				
P54	36.554	30.948	94.091	0.002	0.297
	Composed of: Firm 54 (1)				

For the ESG-F investor, a portfolio concentrated in Firm 3 is achieved in the pre-COVID-19 period. This P3 portfolio is low risk and with an above average expected return (see Table 3).

For the COVID-19 period, thirteen financial efficient portfolios are obtained from among the ESG efficient portfolios of which only two have a positive expected return, namely, P31 (associated with the ESG-inefficient Firm 31) and P54 (associated with the ESG-efficient Firm 54). Therefore, the second type of investor in this period can choose between two portfolios. Both portfolios are high-risk and with expected returns slightly below the mean of renewable energy firms and above those of non-renewable energy firms.

**Fig. 3:**
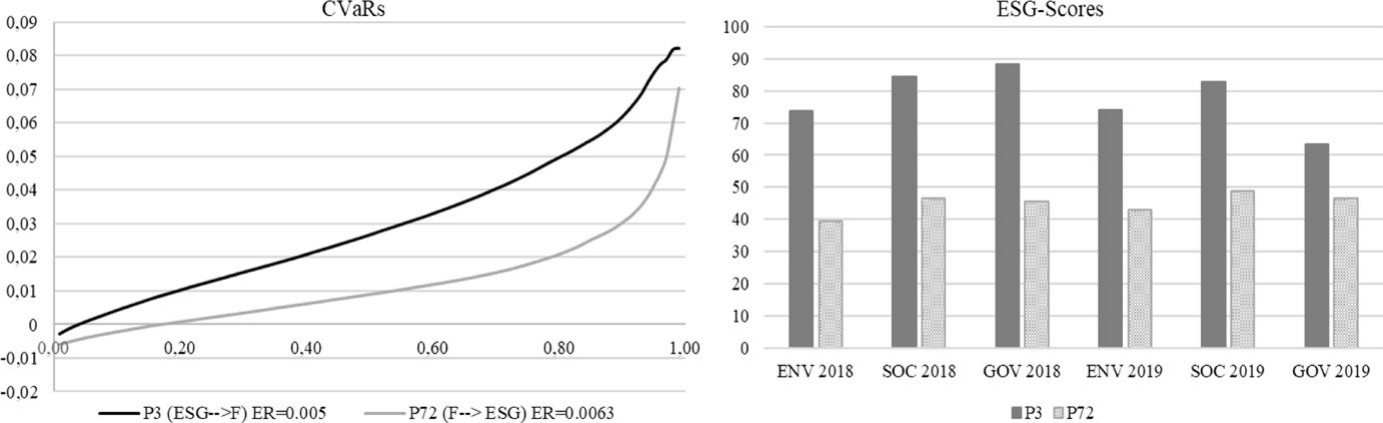
Comparison between portfolios P3 and P72

Lastly, in a similar way to the approach proposed by Perderson et al. (2021), we compared the two types of investors in each period. In the pre-COVID-19 period, for the ESG-F-investor the financial sacrifice incurred by choosing P3 rather than P72 could be measured by the pair, this being composed by the difference between the two expected returns, ER(P72) and ER(P3) and the area between the two $CVaRs$ curves, $CVaRs$(P3) and $CVaRs$(P72), that is ( − 0.019, 0.0223). As we can see in Fig. 3, the portfolio P72 dominates (according to Koopmans-Pareto dominance) the portfolio P3 with respect to financial characteristics. If we compare P72 and P3 with respect to their ESG characteristics we can observe that P3 dominates P72 (see Fig. 3). The ESG sacrifice for the F-ESG investor measured by the differences of the ESG-scores of portfolios P72 and P3, is ( − 34.256, − 37.982, − 42.584) for 2018 and ( − 30.864, − 33.991, − 17) for 2019. From the comparison between P22 and P3 it is possible to conclude that P3 dominates P22 with respect to ESG characteristics and the financial sacrifice is focused on the loss of the expected return.

**Fig. 4:**
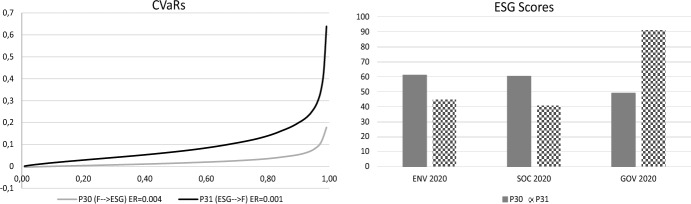
Comparison between portfolios P30 and P31

In the COVID-19 period, the portfolio P30 dominates (according to Koopmans-Pareto dominance) the portfolios P31 and P54 with respect to financial characteristics but these are not comparable with respect to their ESG characteristics. The financial sacrifice of the ESG-F-investor choosing portfolio P31 is ( − 0.03, 0.0675) (see Fig. 4). In this case the ESG sacrifice for the F-ESG investor choosing portfolio P30 is only focused on the GOV-score ( − 41.97).

**Fig. 5:**
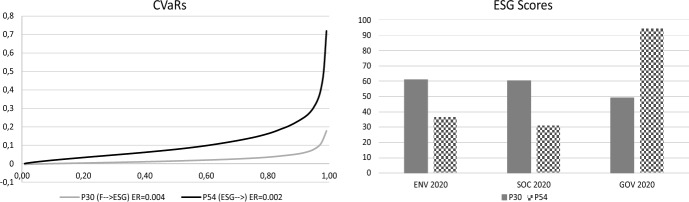
Comparison between portfolios P30 and P54

In the event that portfolio P54 is chosen, the ESG-F investor's financial sacrifice would be ( − 0.002, 0.0822) (see Fig. 5). In this case the ESG sacrifice for the F-ESG-investor choosing portfolio P30 is only focused on the GOV-score ( − 45.093) (see Fig. 5).

#### Out-of-sample analysis of the portfolios’ performance

**Fig. 6:**
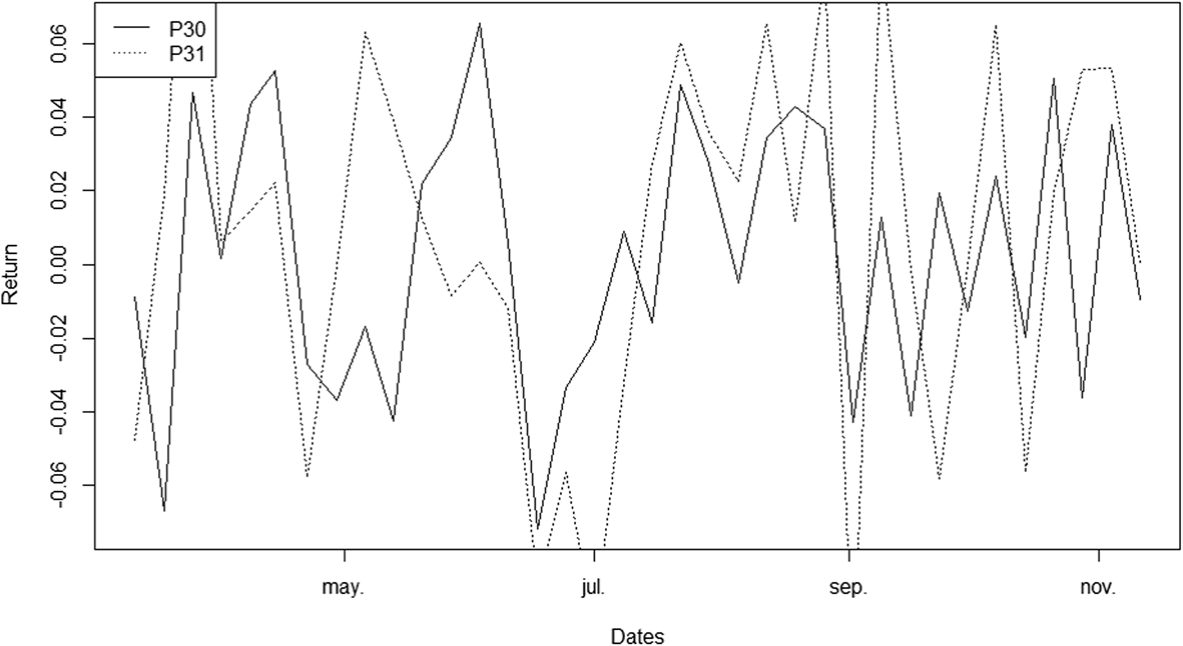
Out-of-sample performance of both the F-ESG and ESG-F portfolios

To study the performance of the portfolios obtained for the F-ESG and the ESG-F investors during an out-of-sample period in which COVID-19 no longer conditions the behaviour of the financial markets, we considered weekly stock prices for each company over the period 3/1/2022–11/11/2022. We choose the portfolios P30 (related to the F-ESG investor) and P31 (related to the ESG-F investor) to carry out an analysis of their performance during this out-of-sample period (see Fig. 6). P30 achieves an expected return equal to 0.003033 and a *CVaR*_95_ equal to 0.0694. Taking into account the financial performance of P30 in the COVID-19 period (*ER* = 0.004, *CVaR*_95_ = 0.075) we can conclude that the behaviour of the portfolio is relatively stable. P30 maintains both a low profitability and low risk profile in the following period. Instead, P31 achieves an out-of-sample (3/1/2022–11/11/2022) expected return equal to 0.0075 and a *CVaR*_95_ equal to 0.106. That is, P31 increases profitability and decreases risk during the new period with respect to the COVID-19 period (*ER* = 0.001, *CVaR*_95_ = 0.256). Therefore, we observe a remarkable improvement in the performance of the ESG-F investor portfolio. For the out-of-sample period, the portfolios related to both the investors’ profiles are not financially comparable. In summary, the out-of-sample ESG-F investor suffers no financial sacrifice.

## Conclusions

A study of the energy stock market in the period 2018–2022 was conducted. For this purpose, two DEA models—financial and ESG—were applied to six data sets obtained from a database composed of 78 firms in the energy sector and their weekly closing prices over the study period. The whole database was divided to take into account two criteria: a temporal criterion (pre-COVID-19 and COVID-19 periods) and energy class (renewable and non-renewable energy).

The financial DEA model was very stringent because it identified only one firm as efficient with the highest expected return in each data set and the average efficiency was low. This is not surprising because SSD dominance is a difficult condition to verify for a single company. The ESG DEA model was introduced with the aim of modelling investors’ preferences and generating the ESG-efficient frontier by moving the weights of radial improvements in ESG scores. If the analysed firm was financially (ESG) inefficient, then the models found a portfolio that strictly dominates the firm and was financially (ESG) efficient at the same time. In consequence, the interest of the approach is that it allows investments that are financial (ESG) efficient in this sector to be identified.

Our findings are interesting for investors, energy policymakers, and for society in general. The results of the analysis confirm the impact of the COVID-19 pandemic on the energy sector worldwide. The financial performance of the renewable energy subsector slightly outperformed that of the non-renewable energy one. With respect to ESG efficiency, although it improved during the COVID-19 period relative to the pre-COVID-19 period, the increase was lower than that of financial efficiency. The financially efficient portfolios contained mostly renewable energy firms (87% during the pre-COVID-19 period and 91.6% during the COVID-19 period).

As another contribution of this paper, a sequential and hierarchical methodology was proposed for investors with both financial and ESG goals. The sequence of applying the two models is determined by the investor’s profile. A conventional investor with ESG concerns could obtain their portfolio by first executing the financial DEA model and then applying the ESG model to the set of financially efficient portfolios. In this way, financially efficient portfolios with “good” behaviour in ESG could be obtained. This type of investor would assume a possible ESG sacrifice that could be measured. On the other hand, an SR investor might choose to first apply the ESG model to generate ESG-efficient portfolios and then the financial DEA model. Naturally, the investor would here be assuming a possible financial sacrifice that could also be measured.

Future research will address the construction of ESG indices from published ESG rating scores. We will try to model the interdependence between ESG criteria and apply thresholds to the levels of ESG performance. This new approach will be introduced in the ESG DEA model. In addition, the proposed methodology can be applied to obtain intersectoral-efficient portfolios.

## Data Availability

The data set generated during the current study is not publicly available as it contains proprietary information that the authors acquired through a license. Information on how to obtain it and reproduce the analysis is available from the corresponding author on request.

## Appendix

**Table 19:** Refinitv ESG pillars and categories

Pillar	Category (components)	Description
Environmental	*Resource Use* (38): water and energy efficiency policies; environmental management systems; total energy and water use; renewable energy use ratio; green buildings; supply chain management and monitoring	Reflects a firm's performance and capacity to reduce the use of materials, energy or water, and to find more eco-efficient solutions by improving supply chain management
	*Emissions* (60): emission policies and targets; total CO2 emissions; climate change opportunities; waste management; e-waste reduction; environmental restoration; staff transportation impact reduction; environmental expenditures	Measures a firm's commitment and effectiveness towards reducing environmental emission in the production and operational processes
	*Innovation* (35): environmental project financing; environmental products; environmental assets under management; Equator principles; clean energy products	Reflects a firm's capacity to reduce the environmental costs and burdens for its customers, and thereby creating new market opportunities through new environmental technologies and processes or eco-designed products
Social	Workforce (75): health and safety policy; training and development policy; diversity; equal opportunities; salary gaps; turnover of employees; flexible working hours	Measures a firm's effectiveness towards job satisfaction, healthy and safe workplace, maintaining diversity and equal opportunities, and development opportunities for its workforce
	Human Rights (9): freedom of association; child labor; political contribuction	Measures a firm's effectiveness towards respecting the fundamental human rights conventions
	Community (23): fair competition; bribery; corruption; business ethics; community involvement; community lending	Measures the firm's commitment towards being a good citizen, protecting public health and respecting business ethics
	Product Responsibility (41): data privacy; customer satisfaction; quality management systems	Reflects a firm's capacity to produce quality goods and services integrating the customer's health and safety, integrity and data privacy
Governance	Management (70):	Measures a firm's commitment and effectiveness towards following best practice corporate governance principles
	Shareholders (39)	Measures a firm's effectiveness towards equal treatment of shareholders and the use of anti-takeover devices
	CSR Strategy (29)	Reflects a firm's practices to communicate that it integrates the economic (financial), social and environmental dimensions into its day-to-day decision-making processes

**Table 20:** ESG-scores of the financial efficiency firms and their associated portfolios

	Pre-COVID-19 period					
	2018			2019		
	ENV score	SOC score	GOV score	ENV score	SOC score	GOV score
F22	34.9527	58.1410	32.1492	33.2043	57.3116	18.3413
P22	73.8119	84.5513	88.2619	73.9746	82.9326	63.5197
F44	0	2.7654	15.0156	0	2.6601	10.9641
P44	90.2668	87.2661	70.5219	87.9117	90.3358	84.1162

	COVID-19 period		
	2020		
	ENV score	SOC score	GOV score
F17	52.6978	27.0224	41.1499
P17	80.5350	80.2670	76.1370
F27	46.0152	32.2073	49.7098
P27	58.2386	65.0330	84.1117

## Footnotes

1 Let $L = - X$ be the loss variable corresponding to the return described by random variable $X$. It is assumed that $E[X] < \infty$. For a fixed level $\alpha$, the value-at-risk *VaR* is defined as the $\alpha$-quantile of the cumulative distribution function $F_{L}$: $\;VaR_{\alpha } = F_{L}^{ - 1} (\alpha )$. The conditional value-at-risk (*CVaR*) is defined as the conditional expectation of $L$, given $L > VaR_{\alpha }$, i.e., ${\mkern 1mu} CVaR_{\alpha } = E[L/L > VaR_{\alpha } ]$.

2 For more information about the companies that make up our database consult the authors.

3 The calculations were carried out using the MachIt program, a free R package (at http://gking.harvard.edu/matchit).

4 See https://www.refinitiv.com/content/dam/marketing/en_us/documents/methodology/refinitiv-esg-scores-methodology.pdf for more information.
